# Current State of Evidence-Based Long-Term Monitoring Protocols for Breast Plastic Surgery Patients

**DOI:** 10.1245/s10434-024-16003-3

**Published:** 2024-08-05

**Authors:** Isabel W. Ho, Anna Chichura, Holly J. Pederson, Brian A. Xavier, Julie Ritner, Graham S. Schwarz

**Affiliations:** 1 Case Western Reserve University School of Medicine, Cleveland, OH USA; 2 Department of Plastic Surgery, Cleveland Clinic, Cleveland, OH USA; 3 Department of Breast Surgery, Cleveland Clinic, Cleveland, OH USA; 4 Department of Subspecialty Gynecology, Cleveland Clinic, Cleveland, OH USA; 5 Department of Radiology, Cleveland Clinic, Cleveland, OH USA

## Abstract

**Background:**

Recommendations for breast surveillance following breast plastic surgery are frequently changing. Establishing guidelines for long-term monitoring protocols may help identify treatable conditions and prevent untoward sequelae. We sought to evaluate the current state of evidence-based long-term monitoring protocols for patients following breast augmentation, reduction, and breast reconstruction.

**Methods:**

Official guidelines from various American societies and international societies were analyzed for alignment in evidence-based recommendations regarding breast surveillance.

**Results:**

The most recent US FDA update recommends magnetic resonance imaging or ultrasound starting 5–6 years after surgery and every 2–3 years thereafter. Discrepancies exist among professional societies: the American Society of Plastic Surgeons (ASPS) aligns with the FDA, while the American Society of Breast Surgeons and American College of Radiology (ACR) find no role for imaging for asymptomatic cases. Ultrasound is first-line for any implant concerns, with MRI if necessary. European societies oppose routine breast implant imaging. Breast reduction patients lack unique screening protocols; monitoring aligns with age and cancer risk factors. Following mastectomy and breast reconstruction, most organizations advocate for annual clinical examinations, with more frequent examinations initially. Evidence suggests that physical examination is sufficient to detect local cancer recurrence, with imaging only indicated if there is concern for recurrence. No surveillance imaging is recommended by the American Society of Clinical Oncology, National Comprehensive Cancer Network, or ASPS; however, ACR recommends mammography for autologous reconstruction only.

**Conclusion:**

Multispecialty and regulatory body alignment may promote provider and patient adherence. Ongoing studies of long-term outcomes are needed to strengthen the level of evidence for monitoring guidelines.

Guidelines regarding long-term implant monitoring or cancer surveillance of patients who have undergone breast plastic surgery are frequently changing. Whether monitoring patients who have a history of surgery for cosmetic or reconstructive purposes, establishing unified guidelines for long-term surveillance of patients with a history of breast surgery may help to standardize care across providers, promote earlier detection of malignancy or high-risk lesions, and identify long-term complications of previous procedures. While there is some evidence in the literature for long-term follow-up of patients with breast implants, there are even fewer evidence-based guidelines in the literature for patients who undergo cosmetic breast surgery without implants. We sought to evaluate the current state of evidence-based long-term monitoring protocols for patients following breast plastic surgery.

## Cosmetic Breast Augmentation

Since the silicone breast implant moratorium for cosmetic surgery was lifted in 2006, the US Food and Drug Administration (FDA) has issued recommendations both in 2006 and 2020 regarding long-term monitoring of breast implants. As a condition of their approval, the FDA now requires implant companies to conduct post-approval studies to monitor long-term performance and safety of silicone breast implants, specifically core post-approval studies, large post-approval studies, device failure studies, focus group studies, annual physician-informed decision survey, and adjunct studies.^1^ Core post-approval studies are designed to follow women for 10 years after implantation to assess long-term clinical performance. Large post-approval studies enroll more patients than core post-approval studies and assess long-term outcomes, as well as identify rare adverse events by following them for 10 years. Device failure studies further characterize the failure of explanted devices, and focus group studies aim to improve patient labeling. Annual physician-informed decision surveys evaluate the distribution of safety information to potential patients. Because the moratorium on cosmetic implants was lifted in 2006, adjunct studies aim to assess the performance and safety of silicone gel implants implanted for reconstruction and replacement of existing implants from 1992 to 2006.^1^

With any breast implant-based surgery, device rupture or leak is an important consideration. Estimates of the incidence of silicone gel implant rupture ranges from 8.7 to 24.2% at 10 years.^2^ While complications following implant rupture are possible, most patients remain asymptomatic and only 30% of gel implant ruptures present with clinically detectable findings on physical examination. However, undetected silicone gel can lead to capsular contracture, breast pain, swelling, granulomas, and lymphadenopathy.^3,4^ As such, there is a clear need for imaging-based screening of asymptomatic implant rupture.^3,5^ Non-contrast magnetic resonance imaging (MRI) remains the gold standard for breast imaging for silicone implant rupture detection due to its high sensitivity for rupture as compared with mammogram or ultrasound, although is not a study designed to detect malignancy.^6^ Table 1 compares the sensitivity and specificity of different imaging modalities at detecting silicone implant rupture based on previous meta-analyses in the literature.^7–13^

**Table 1:** Sensitivity and specificity of different imaging modalities at detecting silicone implant rupture

	Sensitivity (%)	Specificity (%)	PPV (%)	NPV (%)
MRI	64–99	77–96	77–88	83–99
Mammogram	20–82	81–99	50–96	54–97
Ultrasound	64–84	69–93	57–84	58–97

Data derived from references^7–13^

*PPV* positive predictive value, *NPV* negative predictive value, *MRI* magnetic resonance imaging

The 2006 FDA guidelines recommended routine MRI scans for detection of silicone implant rupture, starting 3 years after surgery and every 2 years thereafter.^14^ However, research has indicated that neither providers nor patients followed these recommendations, in part due to the financial cost of MRI and inconsistency in insurance coverage.^15,16^ The cost of MRI ranges from US$2000–$6000, and is often an out-of-pocket expense for a patient after cosmetic breast augmentation.^17–21^ Research by Carr et al.^15^ indicated that only 37.7% of surgeons follow the FDA guidelines, with 55.0% of surgeons not recommending MRI unless clinical issues arise. A survey conducted by Copeland-Halperin et al.^16^ found only 5.9% of patients adhered to FDA guidelines, with only 15.6% of patients getting an MRI at all since surgery.

Given the cost-prohibitive nature of MRI imaging, some have proposed using alternatives such as mammography or ultrasound. Mammography has limited sensitivity in detecting gel implant ruptures, estimated at approximately 20%, due to the homogenous presentation of silicone gel on X-ray imaging.^7^ Ultrasound does not have as high of a sensitivity for implant rupture compared with MRI, with detection estimates in the 60% range, but it is significantly more cost effective.^11,22–25^ Rietjens et al.^6^ found that ultrasound had a negative predictive value of 85%, therefore if the ultrasound findings are negative, MRI may not be necessary. They proposed screening asymptomatic women with ultrasound every year and with MRI every 5 years.

In 2020, the FDA changed their recommendations for asymptomatic silicone implant rupture to include ultrasound or MRI starting 5–6 years after initial surgery and every 2–3 years thereafter.^26^ The American Society of Plastic Surgeons (ASPS) refers to these FDA guidelines on their own website.^27^ Of note, the FDA does not currently have recommendations regarding saline implants, although saline implant rupture is nearly always clinically detectable due to the design of the implant.^28^ The American College of Radiology (ACR) most recently issued guidelines in 2018 regarding breast implant evaluation.^29^ In contrast to the FDA, the ACR stated that there was no clear role for imaging of saline implants or silicone implants in asymptomatic patients, based on meta-analyses with level 1 evidence.^29^ Clinical suspicion for rupture in patients with saline implants is best evaluated with ultrasound, with no role for MRI in the evaluation of saline implants. For suspected abnormalities in patients with silicone implants, the ACR guidelines recommend imaging with ultrasound or non-contrast MRI if there are suspected implant complications. In contrast to both the FDA and ACR guidelines, the British Society of Breast Radiology, Association of Breast Surgery Great Britain & Ireland, and the British Association of Plastic Reconstructive and Aesthetic Surgeons also recommend against routine imaging in asymptomatic patients.^30^ They recommend that ultrasound should be the first-line modality for any implant concerns, with subsequent MRI follow-up if the ultrasound is equivocal or if there are persistent clinical concerns despite normal ultrasound imaging. These findings are summarized and compared in Table 2.

**Table 2:** Summary of guidelines issued by regulatory and professional associations regarding imaging recommendations for surveillance of clinically suspicious symptoms for women who have undergone cosmetic implant-based augmentation

Medical association (year issued)	Recommended surveillance imaging modalities and schedule	Recommended symptomatic imaging modality
FDA (2006)	MRI starting 3 years after surgery and every 2 years thereafter	MRI
FDA (2020)	MRI or ultrasound starting 5–6 years after surgery and every 2–3 years thereafter	Ultrasound or MRI
American Society of Plastic Surgeons (2020)	MRI or ultrasound starting 5–6 years after surgery and every 2–3 years thereafter	Ultrasound or MRI
American Society of Breast Surgeons (2017)	Not indicated	MRI
American College of Radiology (2018)	Not indicated	Ultrasound or MRI
British Society of Breast Radiology (2018)	Not indicated	First line: ultrasound MRI if necessary
Association of Breast Surgery—Great Britain and Ireland (2018)	Not indicated	First line: ultrasound MRI if necessary
British Association of Plastic Reconstructive and Aesthetic Surgeons (2018)	Not indicated	First line: ultrasound MRI if necessary
Royal Australian College of General Practitioners (2020)	Not indicated	Ultrasound
French Health Authority (2014)	Not indicated	Ultrasound

Non-contrast MRI is recommended in this setting

Data derived from references^8,10,26,29,30,101^

*FDA* US Food and Drug Administration, *MRI* magnetic resonance imaging

Mammographic screening should follow the National Comprehensive Cancer Network (NCCN) and ACR guidelines based on age and breast cancer risk.^31,32^ Counseling and risk assessment is crucial in the evaluation of patients undergoing any breast surgery, as patients with elevated risk may require more frequent imaging for cancer screening, and breast imaging specialists must be adept at assessing mammograms with breast implants. However, these guidelines are driven by patient risk factors rather than a prior history of breast surgery.

## Breast Implant-Associated Anaplastic Large-Cell Lymphoma, Breast Implant-Associated Squamous Cell Carcinoma, and Other Lymphomas

With the increasing awareness of breast implant-associated anaplastic large-cell lymphoma (BIA-ALCL), much fervor has arisen over guidelines for patients who have undergone implant-based breast augmentation. Despite the increasing awareness of BIA-ALCL, the current lifetime risk is estimated to be 1:3817–1:30000 for women with textured implants based on the number of reported cases currently.^33^ However, more recent research from Memorial Sloan Kettering suggests a higher incidence of BIA-ALCL than epidemiological reports suggest, with numbers ranging from 1:355 to 1:871.^28,34,35^ As of April 2022, the FDA has received 1130 United States and global medical device reports (MDRs) of BIA-ALCL.^36^ Of all the cases reported to the FDA, 49% of patients presented with seroma, 23% with breast swelling or pain, 13% with capsular contracture, and 11% with peri-implant mass or lump. The median time from implant to diagnosis was 8 years. In 2011, the ASPS/Plastic Surgery Foundation (PSF) and the FDA collaborated to develop PROFILE, a registry used by providers to report cases of BIA-ALCL, SCC, or any lymphoma or cancer found in the breast implant capsule.^37^ The most recent summary in August 2023 found 74.4% of patients presented with seroma, 38.4% with pain, and 28.4% with capsular contracture. The average time from implant to diagnosis was 10.76 years, with a median of 9.75 years.^38^ In patients with a confirmed diagnosis of BIA-ALCL, NCCN guidelines recommend bilateral explantation with bilateral en bloc capsulectomies, regardless of the laterality of presentation.^39,40^

Currently, the FDA does not recommend routine removal of textured implants in patients who have no symptoms. Patients should be educated about the signs and symptoms of BIA-ALCL and monitor the area around the implant for specific changes, but no unique screening or monitoring protocols have been discussed.^41^

Given the relatively recent identification of BIA-ALCL, there are limited studies in the literature, and the FDA itself has only identified fewer than 20 cases of SCC and fewer than 30 cases of non-anaplastic large-cell lymphomas, with only 10 and 12 MDRs received for SCCs and these lymphomas, respectively. The FDA most recently released a statement on 8 September 2022 discussing newly reported cases of squamous cell carcinoma (SCC) and lymphomas diagnosed in the tissue capsule surrounding breast implants.^42^ Goldberg et al.^43^ reported on two patients with long-standing breast implants (>10 years) who presented with acute unilateral breast erythema and swelling. SCC was identified upon routine histopathology of the capsulectomy specimen. A literature search at the time of publication in March 2021 identified six other patients who presented with similar clinical presentations and all had poor outcomes. Whaley et al.^44^ found that extracapsular extension of breast implant-associated SCC was associated with metastatic disease and worse outcomes, although the limited patient cohort makes it challenging to ascertain. Patients who developed metastases did so within the first year.

Other breast implant-associated lymphomas identified within the literature include follicular lymphoma, human herpes virus-associated primary effusion lymphoma, and lymphoplasmacytic lymphoma.^45–47^ One such example that may be mistaken for BIA-ALCL is breast implant-associated diffuse large B-cell lymphoma (BIA-DLBCL). These may have a similar clinical presentation but may be considered in the differential diagnosis if cytology is negative for BIA-ALCL or other T-cell-associated lymphomas. Nevertheless, reports of BIA-DLBCL remain rare, with only six publications in the literature from 2020 to 2023. These publications are all case reports, and the vast majority of cases of BIA-DLBCL were associated with Allergan macrotextured implants. Because of the paucity of data, there are no existing guidelines for management, although previous studies suggest watchful waiting following surgical resection.^48–53^ Despite the limited reports of breast implant-associated malignancy in the literature, the vast majority of patients appear to present with similar symptoms, namely swelling, seroma, tenderness, mass, skin ulceration, and painful capsular contracture.^48–53^ It is thus of particular importance to emphasize to patients with textured implants that delayed seroma formation warrants immediate clinical assessment, as retrospective cohort studies have found that 10% of delayed seromas are associated with BIA-ALCL or other malignancies.^54–56^ The NCCN released guidelines in 2019 to recommend ultrasound as first-line work-up to assess for fluid collection, breast masses, and lymphadenopathy, with MRI recommended if the ultrasound is equivocal.^40^ This underscores the importance of radiologic and histopathological evaluation for any patients undergoing capsulectomy for implant-related symptoms in differentiating between various breast implant-associated malignancies.^57^

## Breast Reduction and Mastopexy

For patients undergoing breast reduction and mastopexy, imaging of the postoperative breast presents its own challenges. There have been differing recommendations for screening mammography following reduction, however more recent evidence does not support unique screening protocols for women undergoing breast reduction surgery.^58,59^ In average-risk patients under 40 years old, it is likely that the harms of routine screening outweigh the benefits, given the low incidence of cancer in the population.^60,61^ Patients at increased risk for breast cancer should adhere to screening guidelines as recommended by the NCCN and the ACR, which may result in age or risk-related imaging (mammography or contrast-enhanced MRI) prior to surgery. For all women aged 40 years and older, preoperative mammography is recommended to assess for occult malignancy.^62^ While some investigators suggest having a postoperative mammogram in the year following surgery, a comparison of patients who have undergone breast surgery and those who have not, noted no significant differences in identified mammographic abnormalities.^63^ Despite the extensive tissue mobilization involved in breast reductions, postoperative screening mammography did not lead to a higher incidence of imaging or diagnostic interventions when compared with nonoperative controls.^64,65^

Breast imaging specialists should be aware of typical postoperative changes. Breast reduction and mastopexy procedures involves removal of tissue and skin, which can lead to alterations in the normal distribution of fibroglandular tissue. On mammography, this can appear as architectural distortion, parenchymal scarring, focal asymmetries, and fat necrosis.^66^ Architectural distortions often appear as having an upward sweeping configuration consistent with the reorganization of tissue,^67^ as well as fibrotic, linear-appearing bands.^68^ Permanent suture also appears on imaging as parenchymal scars with areas of calcification around the nipple areolar complex in 85% of women postoperatively.^69^

One of the most common causes of palpable abnormalities following breast surgery is fat necrosis.^70^ Fat necrosis results following trauma to adipose tissue, including damage caused by manipulation of the breast during breast reduction surgery. Mammographically, fat necrosis may appear as dystrophic calcification, oil cysts, or heterogeneous masses.^66^ With dystrophic calcifications, the risk of malignancy is not increased. Routine screening is often sufficient and the calcifications often evolve to assume a more classic benign appearance.^71^

Mammography is the most cost-effective screening tool for breast cancer. Concerning findings are often evaluated with diagnostic imaging and ultrasound, although the sensitivity of breast MRI is decreased in the postoperative period following breast reduction due to the postsurgical changes that may mask disease.^61^ Tang et al. found the rates of incidental breast cancer in bilateral mammoplasty were 0.16% in women under 40 years of age and 1.47% in women over 40 years of age, on retrospective review from 1990 to 2017, and the initial tumor burdens were all small in their cohort of 39 patients identified to have incidental cancer; the study included 4804 patients. The ASPS does not recommend additional imaging for women who are undergoing or who have undergone elective breast surgery beyond that which is indicated by age and risk factors, unless there is a specific concern.^72^

## Breast Reconstruction

Breast cancer is the most common malignancy affecting women worldwide, accounting for nearly 25% of cancers diagnosed in women.^73,74^ Although increased screening, improved public awareness, and advances in the surgical and medical management of breast cancer have improved survival in breast cancer patients, it is important to be vigilant for locoregional recurrence, which has been estimated to have a 5-year incidence of 3–8%.^75^ An increasing number of patients are electing to undergo breast reconstruction following mastectomy, either via implant-based reconstruction or autologous tissue-based reconstruction. Providers caring for these patients must become adept at identifying possible recurrences given postoperative changes. Current screening guidelines following mastectomy and breast reconstruction are controversial, with conflicting evidence supporting imaging or physical examination alone.^76–78^

Evidence-based clinical practice guidelines from varying professional organizations conflict in their recommendations for surveillance after breast reconstruction. The American Society of Clinical Oncology (ASCO) recommends clinical examinations every 3–6 months for the first 3 years after surgery, every 6–12 months in the fourth and fifth years, and then annually thereafter.^79,80^ The NCCN advises clinical examinations every 3–6 months for the first 5 years and then annually.^31^ Neither organization, nor the ASPS, suggests performing routine screening mammograms or other imaging of reconstructed breasts after mastectomies unless there is clinical concern for recurrence. Evidence suggests that physical examination is sufficient to detect local cancer recurrence. Any abnormal examination findings that are concerning for recurrence should be investigated with imaging (often focused breast ultrasound) in patients who have undergone post-mastectomy implant-based reconstruction or autologous reconstruction.^76,81^ However, the ACR, while in agreement with avoiding screening mammography in patients with implant-based reconstruction, released new guidelines in 2020 for imaging after mastectomy.^82^ They recommended digital breast tomosynthesis or mammography for patients who underwent autologous tissue flap reconstruction and found no evidence to support routine surveillance imaging in patients with a history of implant-based reconstruction. They state that ultrasound may be appropriate only in instances of palpable lumps or clinically significant pain. Table 3 summarizes the various recommendations from the different organizations for clinical examinations, surveillance imaging, and symptomatic imaging.

**Table 3:** Summary of recommendations issued by different professional organizations regarding clinical examination findings and imaging for cancer recurrence in women with implant-based or autologous reconstruction

Organization (year issued)	Clinical examination guidelines	Surveillance imaging recommendations	Symptomatic imaging recommendations
American Society of Clinical Oncology (2016)^72^	Years 1–3: every 3–6 months Years 4–5: every 6–12 months Years 6+: annually	Not recommended (LOE: 2A)	Not specified
National Comprehensive Cancer Network (2022)^31^	Years 1–5: every 3–6 months Years 6+: annually	Not recommended	Not specified
American Society of Plastic Surgeons (2013)^102^	Per ASCO or NCCN guidelines	Not recommended (LOE: NA, Grade D)	Not specified
American College of Radiology (2020)^74^	No recommendation	Implant—not recommended Autologous—annual mammography or digital breast tomosynthesis	Ultrasound

Data derived from^31,72,74,102^

*ASCO* American Society of Clinical Oncology, *NCCN* National Comprehensive Cancer Network, *LOE* level of evidence, *NA* not available

For patients who elect to undergo breast reconstruction with autologous tissue, there remains inconsistency regarding surveillance of reconstructed breast.^77,81,83–88^ Evidence suggests that surveillance mammography of reconstructed breasts has limited utility in detecting cancer recurrence.^86–88^ The ACR released screening guidelines in 2020 for imaging after mastectomy and breast reconstruction suggesting that digital breast tomosynthesis or digital mammography for patients with tissue flaps may be appropriate, but MRI, ultrasound, and positron emission tomography are usually not appropriate.^82^ Ultrasound or other diagnostic imaging is only appropriate for patients experiencing palpable lumps or clinically significant pain on the side of the mastectomy, either with or without reconstruction.

At our institution, MLO single-view mammography is utilized for surveillance imaging in patients who have undergone autologous breast reconstruction. Single-view mammography is more cost effective, less time-consuming, and decreases radiation exposure as compared with traditional mammography. Recent studies have suggested that single-view digital mammography is comparable with two-view mammography in detecting malignancy, particularly in low-density breast tissue.^89^ There have been no studies directly comparing single-view mammography with other imaging for patients who have undergone autologous breast reconstruction, although studies suggest limited sensitivity due to surgical and radiation changes.^90–92^ Postsurgical changes can include architectural distortion, macrocalcifications, masses, or other asymmetries, which can make it challenging to accurately identify local recurrences. At the National Cancer Centre in Singapore, patients undergo surveillance mammography 1 year after autologous breast reconstruction, with either a latissimus dorsi flap or fasciocutaneous flap with a chest wall perforator; imaging is recommended 1 year after surgery and annually thereafter for at least 5 years.^93^ Surveillance mammography after autologous reconstruction has been shown to be both accurate and effective, with characteristic imaging findings that differ from those in patients who underwent standard breast-conserving surgery. In patients with findings that were indeterminate or suspicious for malignancy, spot compression/magnification view mammography and ultrasound were used for further evaluation, with no use for MRI.^93^

One single-institution, 20-year retrospective review of locoregional recurrences, secondary cancers, and atypical breast neoplasms after autologous breast reconstruction found a local recurrence rate of 5.3%.^94^ Another institution conducted a 10-year retrospective review of patients undergoing immediate autologous free flap reconstruction and found a locoregional recurrence rate of 3.25%.^95^ This is comparable with the <10% locoregional recurrence rate reported in the literature.^94^ Yang et al. conducted a meta-analysis comparing the prognosis of breast cancer patients who underwent mastectomy alone and those who underwent mastectomy followed by immediate breast reconstruction, and found no increased risk of recurrence of breast cancer and no statistically significant difference in disease-free survival, with some studies finding that immediate breast reconstruction was associated with improved prognosis.^96^ Bargon et al. evaluated locoregional recurrence in patients undergoing immediate and delayed autologous postmastectomy breast reconstruction and found no difference in recurrence rates.^97^

Locoregional recurrences are more likely to occur in the skin or subcutaneous tissue, and as such are most often detected by physical examination by either the patient or the clinician, with patients commonly reporting symptoms such as palpable masses, tissue irregularity, skin changes, pain, or tenderness.^98–101^ However, in cases where locoregional recurrence is located in the chest wall or deep in the reconstructed breast, detection can be more challenging. In recent years, there has been a shift towards prepectoral implant-based reconstruction, whereas implants were previously placed below the pectoralis muscle, so either the chest wall or subcutaneous recurrences would be superficial to the reconstruction. As more patients undergo prepectoral reconstruction, chest wall recurrences deep to the implant may be more difficult to detect. Locoregional recurrence is associated with a poor prognosis, with 61% survival at 80 months for skin or subcutaneous tissue recurrences, and 45% survival at 80 months for chest wall recurrence.^96^ Shammas et al. found that post-reconstruction imaging was not a predictor of overall survival, with no statistically significant difference in the 5-year survival rate of patients who underwent routine surveillance imaging, imaging for a palpable mass, or no imaging in the postoperative period.^102^

Acellular dermal matrices (ADM) are immunologically inert tissues that have become an increasingly popular adjunct in breast reconstruction. While they may improve aesthetic outcomes and provide structural support, ADM may contribute to contour irregularities or other palpable masses that can cause understandable concern in patients who have had breast cancer. There is a paucity of data on radiologic features of ADM, and even less on management, but prior studies have found that ADM appears as isodense to glandular tissue on mammography, and may resemble postoperative changes, or even hematoma.^103–105^ On ultrasound, ADM may appear as parallel lines with smooth margins with posterior acoustic shadowing, suggestive of folds of ADM that do not warrant further concern.^104–106^ The revascularization of ADM may present both with or without color flow on ultrasound.^105,106^ Of the case reports in the literature, many of the patients who present with palpable masses related to ADM do so without any other systemic symptoms such as fever, swelling, pain, or erythema.^105–108^

Per the recommendations from the ACR, ultrasound is usually appropriate for initial imaging of suspicious findings, with diagnostic digital breast tomosynthesis and diagnostic mammography also being potentially appropriate. While physical examination findings such as skin changes, palpable masses, contour abnormalities, or palpable lymphadenopathy are often due to benign conditions such as fat necrosis, scar tissue, or foreign body reaction to suture, there should be a low threshold for tissue sampling of radiologically suspicious lesions via core needle biopsy or excision biopsy to rule out recurrence.^109,110^ In patients with a history of fat grafting, clinical judgment is necessary to avoid unnecessary procedures. Our group has previously published on fat grafting in breast reconstruction, and noted that 26.5% of patients with a history of fat grafting underwent imaging. In this cohort, 4.2% of patients had distant metastases, none of whom had evidence of locoregional recurrence.  Futher, there was no association between fat grafting and increased locoregional recurrence. These data suggest that comprehensive documentation and serial examinations are appropriate based on time interval following mastectomy and fat grafting, with ultrasound or mammography to assess concerning palpable lesions if indicated.^110^ Other studies have had similar findings of increasing imaging procedures without an impact on 5-year overall survival or locoregional recurrence-free survival.^111–114^

Management of locoregional recurrence is usually achieved with surgical excision of the area of recurrence and reassessment of the axilla with either repeat sentinel node biopsy or complete axillary dissection.^115,116^ In most cases of recurrence following flap reconstruction, the autologous tissue flap is able to be preserved.^100,117^ Wide local excision with negative margins is an oncologically safe approach in this instance and and enables tissue preservation.^109^ A multidisciplinary approach to treatment planning is paramount when recurrent cancer is detected. Surgery is often followed by local chest wall irradiation, and chemotherapy or hormone therapy may be indicated.^116,118^

## Conclusion

The current state of long-term monitoring protocols for patients following breast plastic surgery is complex and lacks consensus. For patients undergoing cosmetic breast augmentation with implants, there are varying guidelines regarding the timing and modality of screening imaging for implant rupture detection. While MRI is considered the gold standard, its cost may limit its accessibility, leading to further exploration of alterative imaging options such as ultrasound and mammography. Recommendations from regulatory and professional associations largely agree on the lack of necessity for routine imaging of asymptomatic patients with breast implants who have undergone mastectomy^8,10,26,29,30,119^ (Table 2). Of note, the increasing awareness of breast-implant associated lymphomas and other breast implant-related malignancies has highlighted the importance of clinical and sonographic evaluation as well as a possible role for histopathological evaluation in the event of implant-related symptoms or capsulectomy.

There are special mammographic considerations in patients undergoing breast augmentation, reduction, and mastopexy. Preoperative screening imaging is warranted for women aged ≥40 years. High-risk women should undergo screening at the age appropriate for their risk level. Postoperatively, mammographic findings post-reduction or mastopexy may be challenging to interpret due to changes in tissue distribution, retained suture, scarring, and fat necrosis. Nevertheless, mammography remains the most cost effective screening tool for breast cancer, and in patients with breast augmentation, is performed with and without implant displacement. Considerations for supplemental screening imaging is given to those with dense breast tissue.

Following breast cancer surgery and reconstruction, surveillance guidelines vary among professional organizations^31,80,82,120^ (Table 3). Most agree that clinical examinations play an integral role in potentially detecting cancer recurrence, but the routine use of imaging is debated. Physical examination alone may be sufficient for detecting local cancer recurrence, with diagnostic imaging and ultrasound of most utilities for symptomatic cases. Recommendations for surveillance of autologous reconstruction are inconsistent, with limited evidence supporting the utility of screening mammography in detecting recurrence. Ultrasound is typically first line for imaging of any suspicious clinical findings, along with diagnostic mammography or tomosynthesis as indicated.

Overall, further research and consensus are needed to establish standardized, evidence-based monitoring protocols for patients undergoing breast plastic surgery. Guidelines that balance the need for effective surveillance with cost and accessibility will be crucial in reducing unnecessary procedures and optimizing patient outcomes.
